# Paroxysmal Atrioventricular Block: A Rare Cause of Cardiac Arrest

**DOI:** 10.7759/cureus.27092

**Published:** 2022-07-21

**Authors:** Arinze N Bosah, Nikos Pappan, Michael Nestasie, Williams Belden, Amit Thosani

**Affiliations:** 1 Department of Internal Medicine, Allegheny Health Network, Pittsburgh, USA; 2 Department of Cardiovascular Medicine, Allegheny Health Network, Pittsburgh, USA

**Keywords:** electrocardiogram, syncope, cardiac arrest, paroxysmal av block, phase 4 block

## Abstract

Paroxysmal atrioventricular block (PAVB) is characterized by a sudden and unanticipated repetitive block of atrial impulses to the ventricles. It is often triggered by supraventricular and ventricular ectopic beats in patients with diseased His-Purkinje system. We present the case of a 69-year-old woman with a history of fascicular block who was admitted with gastrointestinal bleeding. Her hospital course was complicated by cardiac arrest. At the time of the loss of consciousness, telemetry tracings showed sudden onset high-grade second-degree atrioventricular (AV) block with a delayed escape rhythm resulting in a prolonged pause. Adult cardiac life support was necessary along with transvenous pacing until she ultimately underwent the placement of a permanent pacemaker. Thorough evaluation of electrocardiograms (EKGs) and telemetry allowed for accurate diagnoses and appropriate treatment of cardiac arrest secondary to paroxysmal AV block.

## Introduction

Phase 4 heart block or bradycardia-dependent block is one of the postulated mechanisms of pause-dependent atrioventricular (AV) block, particularly in the diseased His-Purkinje system (HPS) [1]. The vast majority of cases present with presyncope and syncopal episodes [2,3], while cardiac arrest is a very rare manifestation. We present a unique presentation of pause-dependent paroxysmal AV block (PD-PAVB) and review its mechanism.

This case was previously presented as a poster presentation at the 2021 American College of Cardiology Annual Scientific Meeting on May 17, 2021.

## Case presentation

**Figure 1:**
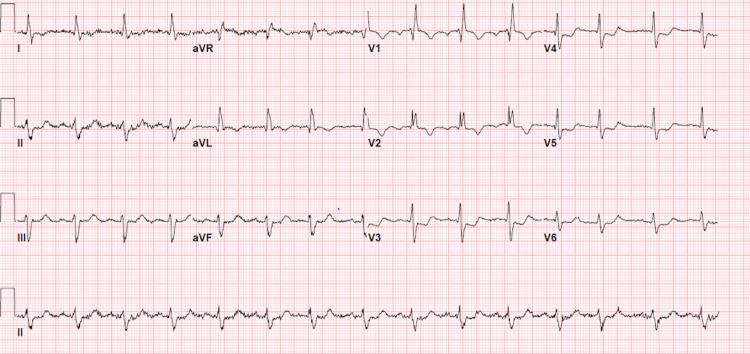
Baseline surface electrocardiogram Baseline electrocardiogram demonstrates normal sinus rhythm with right bundle branch block and left anterior fascicular block. The tracing also shows two PACs: Beats 7 and 13. PACs: Premature atrial contractions.

A 69-year-old woman with a past medical history of diabetes mellitus, paroxysmal atrial fibrillation maintained on coumadin, coronary artery disease status post three-vessel coronary artery bypass grafting, peripheral artery disease, carotid artery disease, right bundle branch block, and left anterior fascicular block (Figure 1) was admitted to the hospital for acute blood loss anemia secondary to gastrointestinal (GI) bleed. She initially presented to an outside hospital with complaints of exertional figure and melanotic stool for the past few days. On presentation to the outside hospital, her vital signs were BP 140/57 mmHg, HR 72 beats per minute, 98.7°F, and saturating 98% on room air. Her labs were pertinent for hemoglobin of 7.9 g/dL (reference range: 12.3-15.3 g/dL), which was a drop from a baseline of 11 g/dL, hematocrit of 23.9% (reference: 36%-45%), platelet of 385,000 (reference: 145,000-445,000), and an international normalized ratio (INR) of 4.1. Her chemistry panel was within normal limits. Iron studies were consistent with iron deficiency. She received 5 mg of vitamin K intravenously (IV) but did not receive packed red blood cell transfusions, given hemodynamic stability.

**Figure 2:**
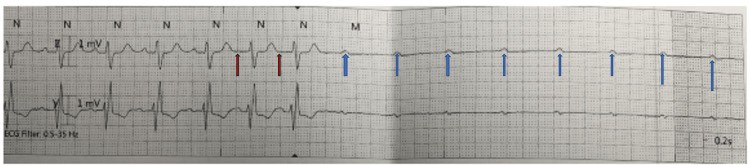
Telemetry rhythm strip prior to the cardiac arrest The rhythm strip demonstrates sinus rhythm with right bundle branch morphology, multiple non-conducted atrial beats (blue arrows) preceded by two premature atrial contractions (red arrows).

A nasogastric (NG) tube was placed, which showed non-bloody gastric secretions. She was subsequently transferred to our hospital for endoscopic evaluation and was started on IV pantoprazole twice daily and IV iron. An upper endoscopy performed on arrival at our hospital showed a small hiatal hernia and hematin in the antrum, which were thought to be due to the NG tube placement trauma, but otherwise, the findings were normal in the esophagus, stomach, and duodenum. She developed hematochezia, and the next day, her hemoglobin was 6.7 and INR was 2.1 requiring transfusion of one unit of packed red blood cells, but she remained hemodynamically stable. The gastroenterologist re-evaluated her and planned for capsule endoscopy and colonoscopy to further investigate the cause of her GI bleed. Overnight, she developed intermittent complete AV block with loss of consciousness (LOC) and profound hypotension requiring several boluses of IV atropine, phenylephrine injections, and brief recurrent rounds of cardiopulmonary resuscitation (CPR). A transvenous pacemaker was placed at the bedside with intermittent loss of capture with associated hypotension and LOC requiring further intermittent CPR. Given persistent relapsing hypotension, she was sequentially placed on dopamine, epinephrine, and norepinephrine continuous drips. She subsequently had a return of native heart rhythm with sinus tachycardia with a heart rate of 110 beats per minute. A review of the telemetry tracing disclosed evidence for PD-PAVB: two premature atrial contractions (PACs) preceding the onset of high-grade second-degree AV block (Figure 2). Laboratory values immediately post-arrest showed normal serum electrolytes, serum creatinine 1.43 mg/dL, lactic acid 6.9 mmol/L, hemoglobin 6.5 g/dL, and leukocytosis to 19 k/mcL. The patient was intubated and maintained on mechanical ventilation for airway protection and transferred to the cardiac intensive care unit. Post-resuscitation electrocardiogram (EKG) showed no new ST-T wave abnormalities. She was transfused two units of packed red blood cells, with an appropriate rise in hemoglobin to 10.7 g/dL. No overt bleeding was noted to suggest hemorrhage as a potential cause of her arrest.

A transthoracic echocardiogram (TTE) was performed and was notable for heart failure with a mid-range ejection fraction (HFmrEF) of 45%-49% and left ventricular regional hypokinesis involving the anteroseptal, apex, and anterior walls. Subsequently, left heart catheterization demonstrated patent left internal mammary artery graft to the left anterior descending artery and patent saphenous vein grafts to the obtuse marginal branch and distal right coronary artery. She was initially started on broad-spectrum antibiotics with IV vancomycin and cefepime, given leukocytosis and the possibility of septic shock, but was discontinued after two days after all of her infectious workup (blood, urine, and sputum cultures) came back negative. Her shock was attributed to cariogenic shock secondary to complete AV block. A transesophageal echocardiogram (TEE) was performed, which was negative for aortic root abscess or endocarditis as a contributing factor to the complete AV block. Lyme's titers were also negative. She underwent capsule endoscopy and colonoscopy, which revealed non-bleeding ulcers in the sigmoid colon with no stigmata of recent bleeding. With no reversible etiologies, PD-PAVB was attributed as the culprit of her cardiac arrest. Our patient’s hemodynamics stabilized over several days, vasopressors were discontinued, and she was extubated easily. A repeat TTE showed normal left ventricle (LV) size, normal ventricular wall motion, ejection fraction (EF) of 50%, and no valvular abnormalities. She underwent the placement of a dual-chamber/permanent pacemaker for the treatment of her PD-PAVB, which she tolerated well. The rest of her hospital stay was uncomplicated, and she is doing well 15 months post-discharge.

## Discussion

PD-PAVB was defined [3] as a sudden, pause-dependent phase 4 AV block, which is characterized by an abrupt change from 1:1 AV conduction to complete heart block. This treatable disease entity is underrecognized, and as a result, its true prevalence remains unknown. PD-PAVB was loosely described in the American College of Cardiology/American Heart Association 2018 guideline [4] as it relates to vagotonic atrioventricular block (AVB) but, debatably, not as its own clinical entity.

Purkinje cells differ from the working myocardial cells in that they have the potential for automaticity, which is normally suppressed by the faster pacemaker activity of the sinoatrial node [5]. In the condition of PD-PAVB, the cells of the diseased His-Purkinje system lose membrane potential to the extent that their sodium channels are inactivated. This results in the observed second-degree block of subsequent sinus impulses [6]. Under these conditions, the tendency for spontaneous diastolic depolarization is augmented. The sodium channel is fairly inactivated at the reduced level of membrane potential at which the spontaneous action potential occurs, rendering slower conduction in phase 4 depolarization [3,6,7]. If the His-Purkinje system has a significant disease such as our patient with evidence of fascicular block (Figure 1), then conduction block of impulse at the end of phase 4 depolarization can lead to a PD-PAVB [1,3]. This is the classic mechanism by which phase 4 heart block occurs. Restoration of conduction requires an appropriately timed PAC or premature ventricular contraction (PVC) that activates the cells at a short cycle length and resets the membrane potential, thereby terminating PD-PAVB [1,3,7,8].

The majority of patients present with presyncopal or syncopal episodes but only rarely present as cardiac arrest as seen in our patient. It is important to distinguish PAVB from vagotonic AV block because the management for both conditions is slightly different. Vagally mediated AVB is defined as any type of AVB mediated by heightened parasympathetic tone [3,4]. The pause associated with PD-PAVB is often preceded by a premature ventricular or supraventricular extrasystole, while vagotonic AV blocks are preceded by significant PR prolongation [3]. Asymptomatic vagally mediated AV blocks are often benign and do not require placement of a pacemaker, while PD-PAVB, regardless of its symptomatology, will require a permanent pacemaker due to its highly unpredictable escape mechanism.

## Conclusions

PD-PAVB is an underrecognized condition, which commonly presents as presyncope and syncope, but on rare occasions, it can be lethal presenting as cardiac arrest. The diagnosis requires a careful review of the EKG and telemetric rhythm strip prior to or during a cardiac arrest. This, in turn, allowed for definitive management with a permanent dual-chamber pacemaker placement in our patient.
